# Peripheral Blood Inflammatory Markers as A Reliable Predictor of Gastric Mucosal Metaplasia Change in the Middle-aged Population

**DOI:** 10.7150/jca.95159

**Published:** 2024-04-23

**Authors:** Chia-Yu Lai, Chieh Lee, Ta-Sen Yeh, Ming-Ling Chang, Yu-Sheng Lin, Tsung-Hsing Chen

**Affiliations:** 1 Department of Management Information Systems, National Pingtung University of Science and Technology, Pingtung, Taiwan.; 2 Department of Information and Management, National Sun Yat-sen University, Kaohsiung, Taiwan.; 3 Department of Gastroenterology and Hepatology, Linkou Medical Center, Chang Gung Memorial Hospital, Taoyuan, Taiwan.; 4 College of Medicine, Chang Gung University, Taoyuan, Taiwan.; 5 Healthcare center, Chang Gung Memorial Hospital, Taoyuan, Taiwan.; 6 Division of cardiology, Department of Internal Medicine, Chang Gung Memorial Hospital Taoyuan Branch, Taoyuan, Taiwan.; 7 Department of General Surgery, Chang Gung Memorial Hospital, Chang Gung University College of Medicine, Taoyuan City, Taiwan.

**Keywords:** intestinal metaplasia, Systemic Inflammation Response Index, Systemic Immune Inflammation

## Abstract

**Purpose:** The study aims to evaluate the efficacy of peripheral blood inflammatory markers as clinical predictors for gastric intestinal metaplasia (IM), a known precursor to gastric cancer. This research investigates the potential of these markers to serve as reliable indicators for detecting gastric IM.

**Methods:** A retrospective cohort study was conducted on 59,143 individuals who underwent checkups at the Taoyuan Chang Gung Memorial Hospital Health Clinic Center from 2010 to 2014. Of these, 11,355 subjects who received gastroscopic biopsies were recruited. After omitting cases with incomplete blood data, the sample was narrowed to 10,380 participants. After exclusion and propensity score matching, subjects in the group with IM and control patients without IM were balanced and included in the study. These subjects were stratified by gender and age, and predictors such as the Systemic Inflammation Response Index (SIRI), Systemic Immune Inflammation Index (SII), and Monocyte-to-Lymphocyte Ratio (MLR) were evaluated. Multivariate logistic regression models were employed to analyze the presence or absence of IM accurately.

**Results:** Out of the 10,380 subjects, 2,088 (20.1%) were diagnosed with IM, while 8,292 (79.9%) did not have IM. In our analysis, inflammation indices were found to have a limited impact on younger patients. For middle-aged and elderly individuals, SII showed statistical significance for predicting IM in males (*p=0.0019*), while SIRI and MLR were significant for females (*SIRI p=0.0001, MLR p=0.0009*). Additionally, the Area Under the Curve (AUC) value indicated that inflammation indices were more influential in females (55.1%) than males.

**Conclusions:** The study results reveal that peripheral blood inflammatory markers could be useful in predicting gastric mucosal metaplasia changes, particularly in middle-aged and elderly populations. Although the markers' predictive power varies with gender, they represent a significant step forward in the non-invasive detection of gastric IM. This could aid in the early identification and management of precancerous conditions.

## Introduction

Gastric cancer (GC), particularly in its advanced stages, continues to pose significant challenges to the healthcare industry due to the scarcity of early detection biomarkers 1, thereby severely affecting survival outcomes. While early detection and intervention in precancerous gastric lesions and stage I GC can significantly enhance the 5-year survival rate to over 95% 2, identification at such early stages remains elusive. GC is classified into two primary histological types according to the Lauren classification: intestinal and diffuse 3-5. The intestinal type predominantly develops from atrophic gastritis (AG) and intestinal metaplasia (IM), with IM identified as a critical transition point. Notably, *H. pylori* eradication has been shown to improve the histological state of the gastric mucosa in AG but not in established IM cases. In the context of gastric IM surveillance, there is yet to be a unified set of guidelines, though annual surveillance is recommended for patients with certain risk factors, including extensive IM, a family history of GC, or lifestyle factors such as smoking 6. Thus, the urgent need for a simple and reliable non-invasive testing method is evident. Systemic immune inflammation indices, such as the Systemic Immune Inflammation Index (SII) and System Inflammation Response Index (SIRI), have gained widespread recognition for their prognostic value in various diseases, including gastrointestinal tract cancer 7, 8. Given their utility in assessing systemic inflammation and immune status, we aimed to investigate their potential application as biomarkers in the context of gastric IM.

This study explores the relationships between peripheral blood inflammatory markers, including the monocyte-to-lymphocyte ratio (MLR), SII, and SIRI, and the presence and progression of gastric IM. By conducting a comprehensive analysis, we aimed to clarify their potential as non-invasive biomarkers for early detection and risk stratification in patients at risk of developing GC. This research may facilitate the development of a more effective surveillance strategy, ultimately improving the prognosis and survival outcomes for individuals at risk of this challenging malignancy.

## Materials and Methods

The methodology was a crucial component of our research, as it lays the foundation for a comprehensive analysis to predict IM. In this section, we provide a detailed explanation of our data selection process, and our inclusion and exclusion criteria. We also provide detailed information regarding our statistical methods and the software used. This study was approved by the Chang Gung Medical Foundation Institutional Review Board (IRB) under protocol number 202300866B0.

### Data collection and preprocessing

**Figure 1:**
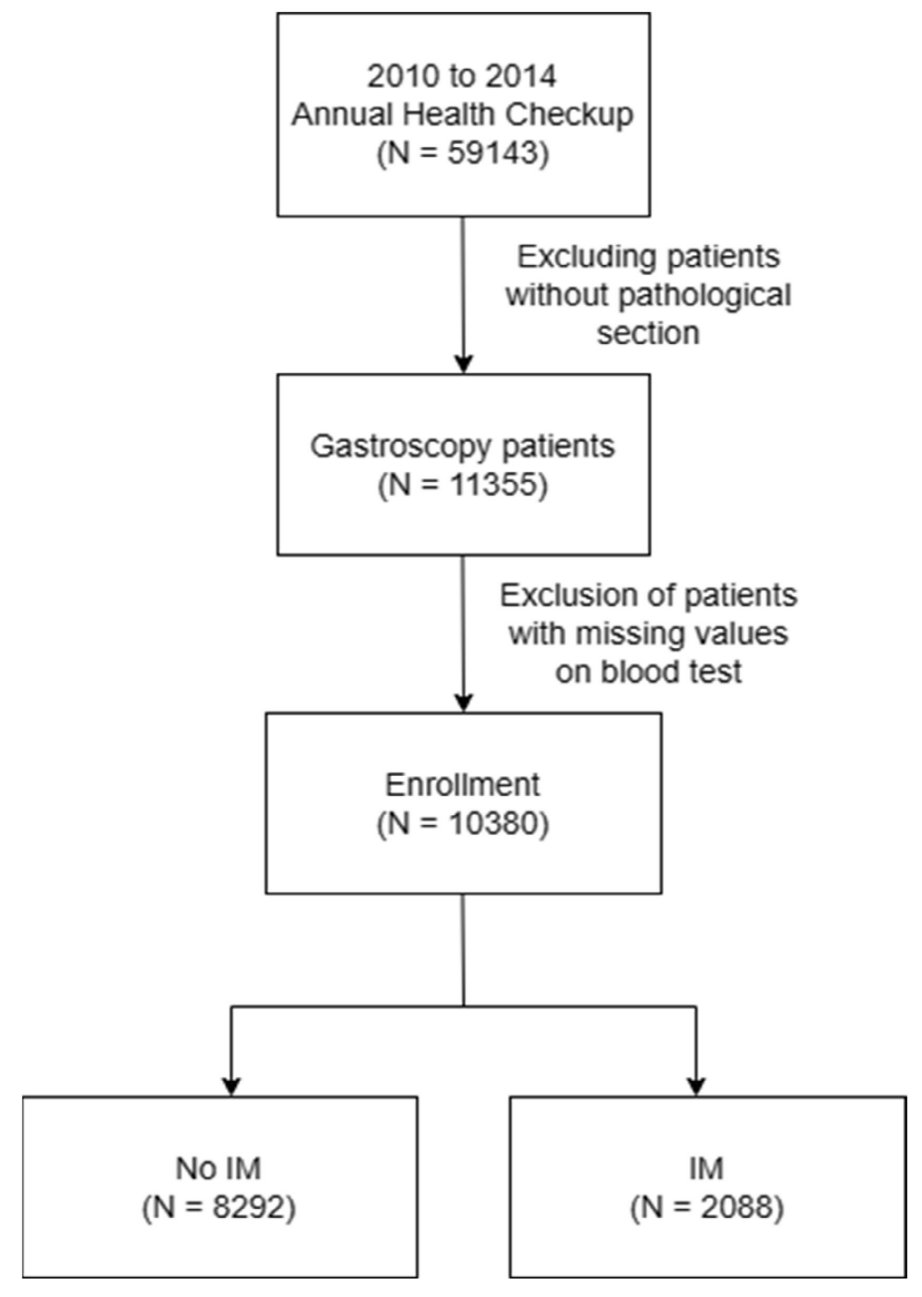
Flow chart of study population selection.

**Figure 2:**
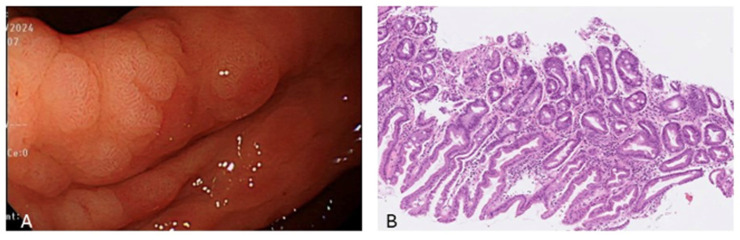
A is the endoscopic view of intestinal metaplasia and B shows microscopic pathology of intestinal metaplasia with IHC stain which presence of scattered goblet-like cells.

From 2010 to 2014, a total of 59,143 subjects were initially included in this retrospective cohort study, 11,355 of whom had undergone endoscopic biopsy and underwent further analysis. After excluding cases with incomplete blood test data, the sample size was reduced to 10,380 subjects. As illustrated in Figure 1, we then separated the subjects into two groups: 2,088 patients with IM, and 8,292 without IM, the pathology with IHC stain shown in Figure 2. This section presents an overview of the study design and methodology used to investigate the IM-related factors. This detailed analysis of the patient data provides valuable insights into IM and may be applied to inform future research and treatment options.

The enrolled criteria at the annual health checkup:

1. Patients underwent upper endoscopic gastric biopsy with a complete blood test.

2. Patients without NSAIDs use it within one week.

The exclusion criteria at the annual health checkup:

1. Patients who are uncooperative, unwilling, or have impaired consciousness.

2. Patients with conditions such as pregnancy or systemic diseases that may affect anesthesia and safety.

3. Individuals who had bleeding or ischemic stroke within the last six months.

4. Those with cardiovascular or pulmonary diseases that pose a risk during the checkup.

5. Patients with abnormal liver function, bilirubin, or platelet levels.

6. Individuals with abnormal thyroid function or poorly controlled diabetes.

7. People who underwent major surgery in the last six months.

8. Individuals with drug or alcohol addiction.

9. Patients with severe ankylosing spondylitis or expected airway difficulties.

10. Obesity with severe obstructive sleep apnea or BMI > 35.

11. Abnormal potassium levels (K < 3.0 or K > 5.0).

12. Nail polish should ideally be completely removed or removed from at least one fingernail on each hand for anesthesia safety. If not removed, anesthesia should be canceled.

The individuals were divided into three groups by age: young (ages 18-45), middle-aged (ages 45-70), and older adults (ages 70+).

From the annual check-up data, we calculated the inflammation indicators, including the MLR, SIRI, and SII based on the following equations:

MLR = 
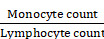


SIRI = 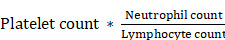


SII = 
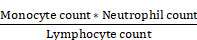


### Statistical analysis

Data analysis was processed using MongoDB database and statistically analyzed using Python packages. For the present study, we employed the t-test to compare two groups and sample proportion test as the univariate analysis method, as well as logistic regression as the multivariate analysis method. All statistical analyses were conducted using Python and with Python's statistical packages, including scipy.stat, ttest_ind, psmpy,and sklearn.linear_model. Our model incorporated a curated set of significant variables in predicting IM. To ensure the reliability of our findings, we employed a dual-faceted analytical strategy.

In the first phase of our analysis, the univariate examination, we utilized the t-test and z-sample proportion test to evaluate the association of each variable with the incidence of IM. We employed Propensity Score Matching (PSM) analysis by using the Python psmpy package, a proficient method for generating a well-matched sample of treated and untreated units that exhibit similar propensity scores. This technique effectively reduces bias and confounding in observational data. We then proceeded to a multivariate logistic regression analysis, executed through a systematic stepwise selection process. We selected the logistic regression model due to its proficiency in managing binary outcome variables, providing a precise measure of the presence or absence of IM. This methodological approach was essential in establishing a scientifically rigorous and statistically sound predictive framework.

To determine the most effective threshold for predicting the occurrence of IM, we conducted a thorough analysis of the inflammation indices using the AUC (Area Under the Curve) method, derived from the Receiver Operating Characteristic (ROC) analyses. The AUC is a benchmark metric that evaluates the efficacy of binary classifiers, such as logistic regression models. This summary measure is a valuable tool for assessing a model's ability to distinguish between two diagnostic groups, namely diseased and normal, thereby providing a comprehensive evaluation that considers all possible classification thresholds. It is a reliable overall performance indicator, offering valuable insight into a model's capabilities. Our analysis aimed to identify the cutoff value that would optimize the Youden index, which is a criterion for selecting the most effective threshold within a sensitivity-favored range. The Youden index is calculated as follows:

Youden Index=Sensitivity+Specificity-1

The cut-off value is then selected where the index is at its highest. Sensitivity, also referred to as the true positive rate, is the proportion of actual positives that are correctly identified, while specificity, also known as the true negative rate, is the proportion of actual negatives that are correctly identified. We evaluated the Youden index for each possible cut-off value, whereby the one with the highest value was selected as the optimal cut-off.

## Results

In this section, we summarize the statistical results from the annual check-up data. The patients' geographical information, including age, gender, and BMI are summarized in Table 1. In addition, the univariate, multivariate analysis results and before propensity score matching and after propensity score matching for all individuals are summarized in Table 2.

The results of the multivariate logistic regression analysis indicate that the inflammation indices (MLR, SIRI, SII) have limited effectiveness in predicting the occurrence of IM, as the AUC obtained was 53.4%. Although we noted a significant relationship between inflammation indices and the occurrence of IM, the relationship was weak for prediction purposes, possibly due to unaccounted for variables, or the multifactorial nature of IM. These study results suggested the need to investigate the impact of inflammation indices on different patient demographics, such as gender and age, to better understand the predictive potential.

Through our analysis of inflammation indices and the AUC, we determined the optimal threshold for predicting the occurrence of IM. Our findings revealed that a cutoff value of 0.513 offered the best balance between sensitivity and specificity, as indicated by the peak of the Youden index. This value strikes a favorable balance between detecting true cases of IM and minimizing false alarms; thus, it presented the preferred threshold for accurately identifying patients at risk for IM while minimizing the misclassification of healthy individuals.

To further investigate the predictability of inflammation indices to IM among different age and gender groups, we separated the patients into female and male subgroups. The AUC for the male and female groups were both 53.7%, although with different significant inflammation indices based on the multivariate analysis. Specifically, SII was significant in the male group, while SII and SIRI were significant in the female group, as shown in Table 3.

The AUC for both male and female subgroups was found to be 53.7%, indicating a similar level of predictability for IM occurrence across genders. However, the multivariate analysis revealed that the significant inflammation index differed between the male and female groups, suggesting that while the overall predictability was similar, the factors underlying this predictability may be different. This implied the need for gender-specific models or cutoff points to optimize the predictive power of the inflammation indices for IM occurrence in the clinical setting.

As shown in Table 3, we found that the inflammation indices differ in terms of effectively classifying IM patients and normal patients based on gender. In addition, previous studies have reported that inflammation does not significantly impact IM development in younger patients 9, 10. Therefore, to clarify the relationship of each inflammation index with IM, we further separated the patients according to age, rendering six different groups. These consisted of male and female groups under 45 years, male and female groups between 45 and 70, and male and female groups over 70 years of age.

The results of our gender and age stratification analyses revealed several notable findings. The group of males aged <45 exhibited an AUC of logistic regression of 51.5%, less than that of the overall patients. Compared to the overall patients' univariate and multivariate analyses results, the inflammation indices exhibited an insignificant impact on male patients <45. As for the female group of the same age, similar results to the male group were noted. The results of two of the three inflammation indices were insignificant, with an AUC of the multivariate analysis of only 50.9%, as shown in Table 4. However, in contrast to the younger male group, our analysis revealed that the SIRI exhibited a significant score for the younger female group, suggesting that the inflammation indices have differing effects based on gender.

In terms of the group of males between 45 and 70 years of age, the only significant inflammation score according to the multivariate analysis was the SII. The AUC of the multivariate analysis was 51.9%, less than the overall patient AUC. This indicates that the discriminability of the inflammation indices for IM in young and middle-aged males is not better than that of the other age and gender groups. In contrast to the males, the same age group of females had an AUC in the multivariate analysis of 55.1%, above that of the overall patients. Our multivariate analysis indicated that all three inflammation indices are significant discriminators of IM for the group of females aged 45 to 70 years, as shown in Table 5.

The AUC of the multivariate analysis for the male group >70 years old was 59.1%, above that of the overall patients. By contrast, the inflammation indices showed an insignificant effect in both the univariate and multivariate analysis for females >70 years old, as shown in Table 6. This result indicates that for elderly males, the inflammation indices had a higher impact on IM development.

## Discussion

The most common subtype of gastric cancer (intestinal) progresses through a recognizable sequence of precancerous stages, including inflammation, atrophy, intestinal metaplasia (IM), dysplasia, and eventual carcinoma formation 11. The gastric epithelium swiftly repairs any injury, resisting acid, NSAIDs, or inflammation. Repair mimics intestinal processes, with proliferative activity at glandular necks. Regenerated mucosa resembles pyloric epithelia, expressing TFF2. In humans, this is termed pseudopyloric mucosa, a reliable marker of oxyntic mucosa repair. In animals, it is known as SPEM. Depending on the cause, its presence can be temporary or permanent. The depth and extent of damage determine if it progresses to intestinal metaplasia. Residual metaplastic mucosa may persist, reflecting gastric atrophy without native gastric mucosa, potentially indefinitely 12. Based on pathological reports and immunohistochemistry staining of gastric tissue samples with at least 30% IM for mucins (MUC1, MUC2, and MUC5AC), we defined gastric mucosal metaplasia changes 13.

Despite being the standard modality, conventional screening methods for gastric IM, such as esophagogastroduodenoscopy (EGD), are often met with patient reluctance due to their invasive nature. Moreover, these methods frequently fail to detect precancerous and early-stage GC lesions, with limitations also evident in technologies, including image enhancement endoscopy (IEE) and conventional computed tomography, which exhibit disappointing sensitivity rates. Meanwhile, currently available serological markers, carcinoembryonic antigen (CEA) and carbohydrate antigen 19-9 (CA19-9)^14^, ^15^, demonstrate limited efficacy with relatively low detection levels. Consequently, there is an urgent need to identify and validate novel serological biomarkers for the early detection of precancerous gastric lesions and GC. In this context, peripheral blood inflammatory markers, including SIRI, SII, and MLR have been reported as new inflammatory markers and prognostic indicators for cardiovascular disease and digestive system malignancies 16-18. The link between inflammation and cancer has been well-established, with chronic inflammation recognized as a significant risk factor for various malignancies, including GC. The progression from chronic gastritis, often due to *H. pylori* infection, to atrophic gastritis, IM, and eventually GC, exemplifies this relationship. Thus, we herein explored the application of these markers to predict precancerous lesions. Our study results indicate that integrating the SII, SIRI, and MLR indices into the GC screening protocol could potentially bridge the current gap in early detection methods, ultimately improving patient management and survival outcomes.

A high monocyte count may indicate a chronic inflammatory state, lymphocytes releasing cytokines and engaging in cytolytic activity, while a low lymphocyte count could reflect an impaired immune surveillance capability, creating an environment conducive to carcinogenesis. The MLR participates in this paradigm as a reflection of the body's inflammatory response, and has emerged as a significant biomarker in the field of oncology, particularly in the prognosis of GC and various cancers 18-24. Indeed, one study has emphasized the role of MLR as a prognostic factor in patients undergoing surgery for GC 21. The SIRI, which is derived from the counts of peripheral neutrophils, monocytes, and lymphocytes, can be a robust and dependable indicator to assess the inflammatory status. Meanwhile, the SII, which is calculated from platelets, neutrophils, and lymphocytes, may serve as a comprehensive indicator for predicting inflammation risks.

Our study results show that the impact of inflammation indices on young patients is limited. This finding is informative and can be viewed positively, as it suggests that younger male patients have different factors influencing the occurrence of IM that may not be captured by inflammation indices alone. This could guide further research into other predictive factors or biomarkers, such as miRNA 25, that are more relevant to this group. Furthermore, understanding that inflammation indices have a lesser impact in this subgroup can prevent unnecessary concerns or interventions based on these indices alone. This suggests the need for a more individualized assessment, which could lead to more appropriate and efficient uses of medical resources. Our study results indicate that in terms of middle-aged and elderly patients, males can rely on SII, while females can rely on SIRI and MLR to indicate the probability of IM. From the value of the AUCs, we find that inflammation indices have a higher impact on females compared with males. Our results demonstrating inflammation index correlations with gastric IM, considered a critical point, provides crucial insights into the early stages of gastric carcinogenesis in middle-aged subjects. Gastric IM, characterized by abnormal cell growth, is a direct precursor to cancerous lesions. The identification of a significant relationship between SIRI, and SII suggests that peripheral blood inflammatory markers could serve as an early warning sign, flagging patients at higher risk for developing GC. Of particular note, relative to current standard screening modalities, obtaining this information via peripheral blood inflammatory markers is faster, more convenient, and cheaper. Interestingly, this finding may also shed light on the potential pathogenic mechanisms at play in GC. The elevated monocyte count could be indicative of a heightened state of local and systemic inflammation, contributing to the milieu that fosters dysplasia. Conversely, the reduced lymphocyte count might signify a weakened immune response, unable to effectively surveil and eliminate emerging dysplastic cells.

In patients with gastric intestinal metaplasia change, we suggest they receive an H pylori infection test and adequate eradication therapy 26. Currently, there is no definite consensus on the endoscopic surveillance of gastric intestinal metaplasia (IM). However, Lee 27 suggest that surveillance intervals can be determined based on the severity of the condition. They propose that surveillance intervals can be determined based on the severity of the Operative Link on Gastric Intestinal Metaplasia (OLGIM). They suggest risk-based surveillance: high-risk (OLGIM III-IV) patients undergo early endoscopy every 2 years, intermediate-risk (OLGIM II) patients every 5 years, while most without IM or focal IM (OLGIM 0-I) may not need surveillance.

## Conclusion

The high correlation of SIRI, SII, and MLR with gastric IM not only confirms the role of the integrated indices as a prognostic marker in gastric cancer but also positions it as a potentially valuable tool for early detection and intervention. This could pave the way for the development of novel strategies for the prevention and management of GC, while emphasizing the need for further research to exploit the full potential of peripheral blood inflammatory markers in clinical practice.

## Funding

This work was supported by grants from Chang Gung Memorial Hospital, Taoyuan, Taiwan (CMRPG3L1811 to THC) and MOHW112-TDU-B-222-124011.

## Figures and Tables

**Table 1:** Descriptive statistics for the checkup population

Variable	Before matching			After Propensity score matching		
	Control N=8292 (95%CI)	P value	IM N=2088 (95%CI)	Control N=2087 (95%CI)	P value	IM N=2088 (95%CI)
**Gender** **(male/female)**	4732/ 3560	0.0000	1447/ 641	1560/ 527	0.0000	1447/ 641
**Age**	51.33±10.9	0.0000	52.9±9.2	53.4±10.8	0.03	52.7±10.5
**MLR**	0.18±0.08	0.06	0.17±0.06	0.17±0.07		0.17±0.07
**SIRI**	10.3±5.89	0.0000	9.93±4.42	9.95±5.0		10.15±5.3
**SII**	8.35±2.27	0.13	8.66±2.17	8.85±2.4	0.0099	8.6±2.3

**Table 2:** The inflammation index univariate and multivariate analysis results of gastric IM in all subjects

Variable	Univariate			Multivariate	
	Control N=8292 (95%CI)	IM N=2088 (95%CI)	P value	OR	P value
**MLR**	0.18±0.08	0.18±0.07	0.84	54.906 (10.369-290.730)	0.0000
**SIRI**	10.3±5.9	10.2±5.4	0.22	0.954 (0.932-0.976)	0.0000
**SII**	8.35±2.3	8.6±2.3	0.0000	1.057 (1.042-1.073)	0.0000

*Cut-off point: 0.513.

**Table 3:** The inflammation indices analysis for males vs. females

Gender	Variable	Univariate			Multivariate	
		Control N=4732 (95%CI)	IM N=1447 (95%CI)	P value	OR	P value
**Male**	MLR	0.18±0.08	0.18±0.08	0.3		
	SIRI	11.0±6.0	10.8±5.9	0.13		
	SII	8.54±2.3	8.7±2.3	0.0004	1.032 (1.014-1.05)	0.0005
		Control N=3560(95%CI)	IM N=641(95%CI)	P value	OR	P value
**Female**	MLR	0.16±0.07	0.15±0.05	0.0000	58.183 (3.387-999.421)	0.0051
	SIRI	9.4±5.6	8.8±3.9	0.0000	0.919 (0.885-0.955)	0.0000
	SII	8.1±2.2	8.3±2.2	0.0003		

*Cut-off point male: 0.505, female: 0.509.

**Table 4:** The inflammation indices analysis results for males vs. females (< 40)

Gender	Variable	Univariate			Multivariate	
		Control N=733 (95%CI)	IM N=157 (95%CI)	P value	OR	P value
**Male**	MLR	0.18±0.07	0.18±0.06	0.3		
	SIRI	10.8±5.2	10.4±4.2	0.1		
	SII	8.0±2.0	8.3±1.7	0.001	1.099 (1.039-1.163)	0.001
		Control N=534 (95%CI)	IM N=81 (95%CI)	P value	OR	P value
**Female**	MLR	0.17±0.07	0.17±0.05	0.7		
	SIRI	10.2±5.74	9.9±3.6	0.44		
	SII	7.6±1.9	7.4±1.7	0.05		

*Cut-off point male: 0.509, female: 0.

**Table 5:** The inflammation indices analysis results for middle-aged males vs. females (45-70)

Gender	Variable	Univariate			Multivariate	
		Control N=3752 (95%CI)	IM N=1213 (95%CI)	P value	OR	P value
**Male**	MLR	0.2±0.08	0.2±0.08	0.83		
	SIRI	10.8±5.95	10.7±5.8	0.46		
	SII	8.6±2.3	8.8±2.3	0.002	1.032 (1.012-1.053)	0.0019
		Control N=2841 (95%CI)	IM N=523 (95%CI)	P value	OR	P value
**Female**	MLR	0.16±0.07	0.15±0.05	0.0000		
	SIRI	9.2±5.5	8.5±3.7	0.0000	0.972 (0.958-0.986)	0.0001
	SII	8.16±2.2	8.5±2.2	0.0000	1.044 (1.018-1.072)	0.0009

*Cut-off point male: 0.522, female: 0.513.

**Table 6:** The inflammation indices analysis results for elder males vs. females (>70)

Gender	Variable	Univariate			Multivariate	
		Control N=247 (95%CI)	IM N=77 (95%CI)	P value	OR	P value
**Male**	MLR	0.24±0.1	0.2±0.08	0.002		
	SIRI	14.5±7.8	12.85±6.5	0.009		
	SII	8.8±2.8	9.7±2.7	0.0004	1.125 (1.053-1.203)	0.0005
		Control N=185 (95%CI)	IM N=37 (95%CI)	P value	OR	P value
**Female**	MLR	0.17±0.08	0.17±0.05	0.74		
	SIRI	10.6±6.6	10.23±4.0	0.5		
	SII	8.65±2.6	8.35±2.2	0.25		

*Cut-off point male: 0.483, female: 0.
